# Targeting Gut Microbiota to Combat Vascular Aging and Cardiovascular Disease: Mechanisms and Therapeutic Potential

**DOI:** 10.3390/nu17172887

**Published:** 2025-09-06

**Authors:** Jian Li, Yadong Wang, Sanjiv Shrestha, Andrew T. Gewirtz, Ye Ding, Jun Zou

**Affiliations:** Institute for Biomedical Sciences, Georgia State University, Atlanta, GA 30303, USA; jli73@gsu.edu (J.L.); ywang172@student.gsu.edu (Y.W.); sshrestha7@student.gsu.edu (S.S.); agewirtz@gsu.edu (A.T.G.)

**Keywords:** vascular aging, cardiovascular diseases, gut microbiota, gut-derived metabolites, natural products

## Abstract

Vascular aging, characterized by arterial thickening, reduced elasticity, and endothelial dysfunction, significantly compromises vascular health and accelerates the progression of cardiovascular diseases (CVDs). Emerging research highlights the gut–vascular axis as a critical mediator of vascular health, with the gut microbiota (GM) playing a pivotal role in modulating vascular aging and CVDs. This review presents a thorough and up-to-date discussion of the fundamental mechanisms driving vascular aging and explores how GM and its metabolites influence these processes. Furthermore, we place particular emphasis on therapeutic strategies, including probiotics and food-derived natural products, that foster beneficial bacterial growth and support cardiovascular health, while also exploring the underlying mechanisms. By targeting GM composition and function, these approaches offer promising strategies to mitigate vascular aging and lower CVD risk. Future studies aimed at deepening our understanding of the interplay between GM, its metabolites, and vascular health could pave the way for novel preventive and therapeutic interventions against vascular aging and CVDs.

## 1. Introduction

Cardiovascular diseases (CVDs) encompass a wide range of heart and blood vessel disorders, such as coronary artery disease, stroke, rheumatic heart disease, and other vascular conditions. They remain the leading cause of death and disability worldwide, accounting for an estimated 17.9 million deaths each year—approximately one-third of all global deaths [1]. According to the reports from Centers for Diseases Control, 919,032 people died from CVDs in 2023. The economic burden is also substantial, with heart disease costing an estimated $417.9 billion between 2020 and 2021, including expenses for medical care, prescription drugs, and productivity losses. Vascular aging, characterized by arterial thickening, plaque formation, and reduced elasticity, contributes significantly to the rising prevalence of CVDs and accelerates the progression of conditions such as coronary heart disease, hypertension, and stroke [2,3]. Recent research has highlighted gut microbiota (GM) as a critical factor in vascular health, with dysbiosis contributing to the onset and progression of various CVDs [4,5]. Imbalances in gut bacteria can promote vascular aging, while beneficial microbes play a protective role in cardiovascular health.

Probiotics from beneficial bacterial strains like *Lactobacillus*, *Bifidobacterium*, and *Faecalibacterium* show promise in mitigating vascular aging and CVDs [6,7,8]. These probiotics enhance immune function, reduce inflammation, and improve endothelial health. Additionally, natural products, such as dietary fibers and phytochemicals, support cardiovascular health by modulating the GM composition and maintaining intestinal balance [9]. Targeting the GM with these compounds offers a potential strategy to combat vascular aging and reduce CVD risk in aging populations. This review examines the role of GM in vascular aging and CVDs, emphasizing the therapeutic potential of probiotics and natural products.

## 2. Mechanisms of Vascular Aging and CVDs

Vascular aging is a complex biological process characterized by structural and functional changes in the blood vessels that occur with chronological and biological aging, which leads to increased stiffness, endothelial dysfunction, and a higher risk of cardiovascular diseases [10]. While these changes are age-related, they can be accelerated by pathological conditions such as chronic inflammation, oxidative stress, and metabolic disorders. Understanding these underlying mechanisms of vascular aging is crucial before exploring the potential role of GM in vascular aging. In the following sections, we will discuss the key mechanisms contributing to vascular aging.

### 2.1. Inflammation and Oxidative Stress

**Figure 1:**
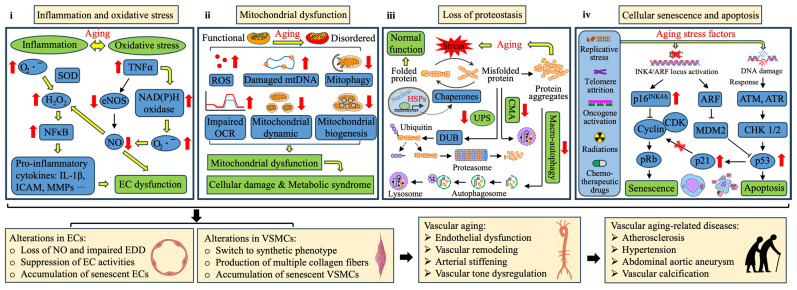
This model predicts that the mechanisms of vascular aging mainly include (**i**) inflammation and oxidative stress, (**ii**) mitochondrial dysfunction, (**iii**) loss of proteostasis, and (**iv**) cellular senescence and apoptosis, all potentially modulated by gut microbiota. Through these shifts in biological function with aging, structural and functional alterations occur in vascular cells (ECs and VSMCs), which result in vascular aging and age-related vascular diseases. (**i**) During aging, the increased level of O_2_^−^ generated by the electron transport chain in senescent EC are dismutated to H_2_O_2_ in mitochondria by SOD. The excessive H_2_O_2_ contributes to the activation of NFκB, resulting in an accumulation of pro-inflammatory cytokines. On the other hand, up-regulation of TNFα in senescent EC, at least in part, promotes the production of O_2_^−^ by the NAD (P)H oxidase and down-regulates eNOS, which is responsible for the impaired bioavailability of NO and endothelial dysfunction. (**ii**) Vascular aging is associated with progressive mitochondrial dysfunction that occurs due to excessive ROS generation, accumulation of damaged mtDNA, impaired oxygen consumption rate, and suppressed mitochondrial activities (mitochondrial dynamic, mitochondrial biogenesis, and mitophagy). The disordered mitochondria accumulated in vascular cells, leading to cellular damage and metabolic disorders. (**iii**) Aging stress on the vascular system triggers increased demand for protein folding, whereas the proteotasis system, including ubiquitin-proteasome system (UPS), chaperone-mediated autophagy (CMA), and macro-autophagy, are significantly declined with aging, which causes the accumulation of aggregated proteins and thus aggravates aging stress. (**iv**) A verities of external aging stress factors, including replicative stress, telomere attrition, oncogene activation, radiations, and chemo-therapeutic drugs, can trigger the exit of senescence-associated cell cycle, which is regulated with the initiation of p16^INK4a^/Rb and p53/p21 pathways. Idea adapted from [19].

Vascular inflammation, or “inflammaging”, is a hallmark of vascular aging, characterized by elevated pro-inflammatory cytokines such as Tumor Necrosis Factor alpha (TNF-α) and interleukins (IL-1β, IL-6, IL-8, IL-13, IL-18) [11]. This leads to endothelial dysfunction and impaired vasodilation in aging arteries [12] (Figure 1i). A major driver of vascular inflammation is oxidative stress, where reactive oxygen species (ROS) react with nitric oxide (NO), reducing its availability and impairing vasodilation. ROS also trigger lipid peroxidation, DNA damage, and protein oxidation [13,14]. A key consequence of oxidative stress is the age-related decline in NO bioavailability, driven by multiple factors, including increased arginase activity, which depletes L-arginine (the substrate for NO synthesis), and the loss of tetrahydrobiopterin (BH4), an essential cofactor for nitric oxide synthase (NOS) [15,16]. Oxidative stress activates Nuclear Factor kappa B (NFκB), upregulating pro-inflammatory genes and matrix metalloproteinases (MMPs), thereby compromising extracellular matrix integrity and vascular homeostasis [17]. The NLR family pyrin domain containing 3 (NLRP3) inflammasome further amplifies inflammaging by promoting IL-1β and IL-18 production [18].

### 2.2. Mitochondrial Dysfunction

Vascular aging is associated with impaired mitochondrial function, resulting in reduced electron transport efficiency and ATP synthesis, which contribute to aging-related vascular diseases [20,21]. A major contributor to this dysfunction is the excessive production of mitochondrial reactive oxygen species (mtROS) by pro-oxidative enzymes such as NADPH oxidase (NOX) [22,23]. Elevated mtROS depletes antioxidants, damages mitochondrial DNA (mtDNA), and disrupts mitochondrial homeostasis (Figure 1ii). The depletion of antioxidants like glutathione (GSH) and Mn-SOD occurs due to peroxynitrite-mediated inhibition, weakening oxidative defense mechanisms [24]. Simultaneously, the Nrf-2 signaling pathway, crucial for antioxidant responses, is compromised, further exacerbating oxidative stress [9]. Notably, the deletion of p66Shc has been shown to reduce oxidative stress and mitigate endothelial dysfunction, highlighting its role in vascular aging [25].

Aging cells also exhibit high mtDNA mutation rates, which drive increased ROS production, apoptosis, and senescence [26,27,28]. Additionally, mitochondrial biogenesis is impaired, as indicated by reduced levels of PGC-1α and TFAM, key regulators of mitochondrial function [29,30]. Dysfunctional mitochondria accumulate in aged vascular cells due to insufficient mitophagy, imbalanced fission-fusion dynamics, and decreased oxygen consumption [31,32,33]. The decline of SIRT1, a key regulator of mitochondrial quality control, further exacerbates these age-related mitochondrial impairments [34].

### 2.3. Loss of Proteostasis

Proteostasis ensures proper protein synthesis, folding, and degradation through molecular chaperones and systems like the ubiquitin-proteasome system (UPS) and autophagy [35]. With aging, this network malfunctions, leading to misfolded protein accumulation and cytotoxic aggregates (Figure 1iii) [36]. Molecular chaperones, such as heat shock proteins (HSPs), prevent protein aggregation, particularly under stress [37,38,39]. Hsp60 increases in atherosclerosis, while mitochondrial Hsp90 accumulation is linked to pulmonary hypertension and vascular remodeling [40,41]. However, Hsp70 inducibility declines in aging aortic tissues, potentially impacting vascular health [42,43,44]. The UPS and autophagy pathways degrade misfolded proteins, but their function declines with age [45]. While proteasome activity is elevated in atherosclerotic plaques as a compensatory response, it eventually decreases, contributing to vascular cell senescence, oxidative stress, and inflammation [46,47]. Dysregulated autophagy, including mitophagy, further accelerates vascular aging, as deletion of key autophagy-related genes increases susceptibility to endothelial dysfunction [48,49,50,51]. Maintaining proteostasis is therefore essential for vascular health, and its disruption plays a significant role in vascular aging.

### 2.4. Cellular Senescence, Apoptosis, and Necroptosis

Vascular cells undergo senescence due to chronic stressors like DNA damage, oxidative stress, and telomere dysfunction, contributing to disease progression through the pro-inflammatory senescence-associated secretory phenotype (SASP) (Figure 1iv) [52,53]. Senescence markers p16, p21, and p53 are elevated in atherosclerotic plaques of older adults [54,55]. Senescent endothelial cells (ECs) increase oxidative stress and reduce NO bioavailability, leading to endothelial dysfunction, impaired vascular tone, and mitochondrial dysfunction [56,57]. Similarly, vascular smooth muscle cell (VSMC) senescence promotes atherosclerosis, hypertension, and diabetes by increasing arterial stiffness, inflammation, and vascular calcification [58,59]. Aging also disrupts programmed cell death, including apoptosis and necroptosis [60]. Aged ECs show impaired NO synthesis and heightened apoptosis, while in vascular injury, increased VSMC proliferation resists apoptotic signals, leading to excessive neointima formation [61]. Necroptosis, marked by cell rupture and DAMP release, activates inflammation and is linked to atherosclerotic plaques and AAA, playing a key role in inflammaging [62,63,64,65,66,67,68].

## 3. Key Mediators of Gut–Vascular Communication in Vascular Aging and Cardiovascular Health

**Figure 2:**
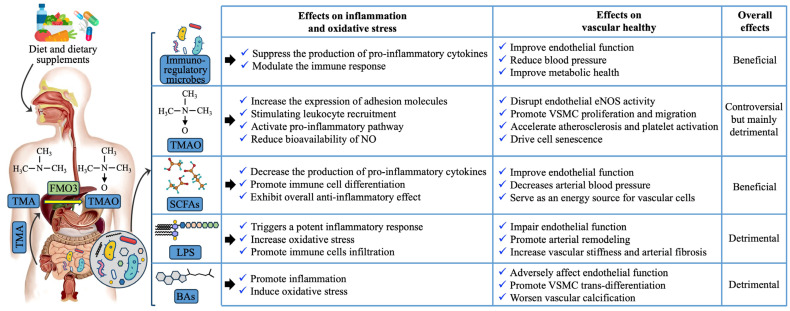
This model predicts the effects of Immunoregulatory microbe and GM metabolites, including TMAO, SCFAs, LPS, and BAs, on inflammation and oxidative stress as well as how they affect vascular health.

The GM influences the cardiovascular system, often through direct communication via microbial signals crossing the intestinal epithelium. These signals include structural components like lipopolysaccharides (LPS) and peptidoglycans, which interact with host cells via pattern recognition receptors, as well as metabolites such as trimethylamine N-oxide (TMAO), short-chain fatty acids (SCFAs), and bile acids (BAs). In the following, we will explore how these microbial components and metabolites influence vascular aging by modulating key factors such as inflammation, oxidative stress, and mitochondrial function (Figure 2).

### 3.1. Trimethylamine N-Oxide (TMAO)

TMAO is a metabolite produced by GM from dietary nutrients such as choline, phosphatidylcholine, and L-carnitine. Certain gut bacteria, including *Desulfovibrio* and *Clostridium* species, harbor trimethylamine (TMA) lyases that convert these dietary precursors into TMA [69]. TMA is absorbed into the bloodstream and oxidized to TMAO by hepatic flavin monooxygenase enzymes, primarily FMO3 [70].

Elevated TMAO levels are strongly linked to CVDs, contributing to inflammation, oxidative stress, endothelial dysfunction, atherosclerosis, VSMCs proliferation, and platelet activation [71,72,73,74,75]. Specifically, TMAO promotes vascular aging by disrupting endothelial nitric oxide synthase (eNOS), stimulating leukocyte recruitment, and increasing adhesion molecule expression, leading to impaired vasodilation and vascular remodeling. TMAO causes mutations in mitochondrial DNA, disrupting oxidative phosphorylation (OXPHOS), raising ROS levels, and lowering ATP/ADP ratios. This results in mitochondrial dysfunction, increased cell death [76]. Additionally, TMAO influences cholesterol and bile acid metabolism and enhances foam cell formation via scavenger receptors like cluster of differentiation 36 (CD36) and steroid receptor RNA activator 1 (SRA1) on macrophages [77].

Although TMAO is closely associated with adverse cardiovascular events, its role as a causative factor versus a biomarker remains debated. TMAO has been shown to amplify angiotensin II-induced hypertension but does not affect normal blood pressure [78], leaving its causative role in question. Despite this, targeting TMAO pathways is under exploration as a potential therapeutic strategy to mitigate vascular aging and cardiovascular risk.

### 3.2. Short-Chain Fatty Acids (SCFAs)

SCFAs are produced by gut bacteria like *Faecalibacterium prausnitzii*, *Bifidobacterium*, and *Akkermansia muciniphila* during the fermentation of fiber. The main SCFAs, acetate, propionate, and butyrate, have emerged as key modulators in preventing and managing vascular aging and its associated CVDs. These metabolites exert their effects through various biological processes, including anti-inflammatory and antioxidant activities, regulation of endothelial function, blood pressure control, and mitochondrial health.

SCFAs help mitigate the chronic inflammation and oxidative stress that characterize vascular aging. By activating free fatty acid receptors (FFA2 and FFA3), SCFAs modulate immune cell function, promoting anti-inflammatory responses [79]. These receptors, found on immune cells like dendritic cells, macrophages, and T cells, help decrease pro-inflammatory cytokine production and foster regulatory immune cell phenotypes, reducing the inflammatory environment in the vasculature. Butyrate, in particular, is known to inhibit histone deacetylases (HDACs), which modulate gene expression related to inflammation and oxidative stress, further dampening inflammatory pathways and promoting vascular health [80,81,82].

In addition to their anti-inflammatory properties, SCFAs also support mitochondrial function and endothelial health. Butyrate enhances endothelial NO production [80], which is crucial for vascular dilation and overall endothelial function. Propionate has been linked to a reduction in blood pressure, especially in hypertensive models [83]. Furthermore, SCFAs like acetate provide energy to vascular cells [84,85], potentially aiding in the maintenance of cellular function and reducing age-related vascular decline.

### 3.3. Lipopolysaccharide (LPS)

LPS, produced by Gram-negative bacteria such as *Escherichia coli*, *Bacteroides fragilis*, and *Enterobacter cloacae*, links GM to vascular aging by driving chronic inflammation, oxidative stress, and cellular dysfunction. Elevated LPS levels activate TLR4 on ECs, immune cells, and VSMCs, leading to ROS production and pro-inflammatory cytokines [86,87]. These responses impair mitochondrial function, disrupt proteostasis, and induce cellular senescence.

LPS-triggered inflammation promotes endothelial dysfunction by reducing NO bioavailability, increasing leukocyte adhesion, and inducing vascular stiffness and fibrosis [88,89]. Oxidative stress from LPS further exacerbates mitochondrial dysfunction, promoting apoptosis and necroptosis in vascular cells, accelerating tissue damage. Recruitment of monocytes and macrophages into arterial walls under LPS influence contributes to foam cell formation and atherosclerosis [90,91]. LPS also activates platelets, increasing thrombotic risks [92,93], a hallmark of vascular aging and CVDs. Targeting GM producing LPS, such as *Escherichia coli* and *Bacteroides fragilis*, offers potential therapeutic strategies against vascular-related diseases.

### 3.4. Bile Acids (BAs)

BAs are amphipathic cholesterol metabolites produced in the liver and modified by GM, including *Clostridium*, *Bacteroides*, and *Lactobacillus*. These bacteria transform primary BAs into secondary BAs such as deoxycholic acid (DCA) and lithocholic acid (LCA), which significantly influence vascular aging.

Several mechanisms link BAs to vascular dysfunction, primarily through inflammation, oxidative stress, and cellular damage. In ECs, BAs such as chenodeoxycholic acid promote ROS production, activating NF-κB and p38 MAPK signaling pathways, leading to increased adhesion molecule expression and inflammation [94]. Hydrophobic BAs exacerbate oxidative stress in VSMCs, causing tissue damage and remodeling [95].

GM-modulated BAs also affect vascular calcification and cardiac function. Hydrophobic BAs like DCA contribute to vascular calcification by inducing VSMCs trans-differentiation through calcium-phosphate metabolism. Elevated BA levels disrupt vascular tone via nuclear and G-protein-coupled receptors, including the farnesoid X receptor (FXR) and Takeda G protein-coupled receptor 5 (TGR5), with detrimental effects on endothelial function and arterial stiffness [96]. Moreover, chronic exposure to high concentrations of hydrophobic BAs, influenced by dysbiosis, increases arrhythmia risk by modulating ion channels in cardiac cells and altering electrical excitability [97]. These effects are further amplified by the inflammatory and oxidative stress environment driven by BA dysregulation, contributing to the progression of atherosclerosis and heart failure.

In conclusion, the interplay between GM and vascular aging forms a dynamic system where microbial metabolites and components significantly impact cardiovascular health. Gut-derived metabolites such as TMAO, SCFAs, LPS, and BAs modulate key factors like inflammation and oxidative stress, playing dual roles in either accelerating or mitigating vascular aging processes.

## 4. Utilization of Beneficial Gut Bacteria as Probiotics in Mitigating Vascular Aging and Enhancing Vascular Health

GM consists of beneficial bacteria that naturally reside in the human gut and play a role in mitigating vascular aging. Some of these bacterial strains are now being investigated as probiotics with potential to support vascular health. These probiotics offer direct benefits, such as immune modulation, anti-inflammatory effects, maintenance of endothelial function, regulation of blood pressure, and improvements in metabolic health. A summary of these probiotic strains and their effects is presented in Table 1 [98,99,100,101,102,103,104,105,106,107,108,109,110,111,112,113,114,115,116,117,118,119,120,121,122,123,124,125,126,127,128,129,130].

Specific probiotics, such as Lactobacillus, Bifidobacterium, and *Faecalibacterium* strains, modulate immune responses by balancing pro-inflammatory and anti-inflammatory activities, helping to alleviate vascular aging [104,131,132,133,134,135,136,137]. For example, *Lactobacillus plantarum* 299v supplementation reduces IL-8, IL-12, and leptin levels, independent of traditional risk factors and TMAO changes [104]. *Bifidobacterium bifidum* PRL2010 increases IL-12 in the kidneys and reduces IL-10 in the liver and kidneys in ischemia/reperfusion injury models [138], while *Faecalibacterium prausnitzii* reduces CD45-positive leukocytes in subcutaneous adipose tissue [139]. Certain *Lactobacillus* and *Bifidobacterium* species also enhance endothelial function and regulate blood pressure. *Lactobacillus helveticus*, for example, lowers blood pressure in hypertensive individuals by producing bioactive peptides that inhibit ACE [121,140,141]. Probiotics also show potential for improving metabolic health, which can reduce vascular aging and cardiovascular diseases. *Lactobacillus casei* improves lipid profiles by lowering total cholesterol, LDL cholesterol, and triglycerides [142]. *Lactobacillus plantarum* enhances insulin sensitivity, glucose metabolism, and lipid profiles [143,144], while *Lactobacillus acidophilus* improves insulin sensitivity and reduces total and LDL cholesterol [145,146,147,148]. *Bifidobacterium breve* and *Bifidobacterium lactis* benefit glucose metabolism and insulin sensitivity [149,150,151].

## 5. Nutrient-Derived Natural Products for Modulating GM and Mitigating Vascular Aging

Nutrient-derived natural products have been integral to managing chronic diseases, including CVDs, due to their diverse pharmacological properties [108,152,153]. Functional foods rich in bioactive compounds provide cardioprotective benefits by mitigating vascular aging through mechanisms such as anti-inflammatory and antioxidant effects, improving endothelial function, regulating blood pressure, and optimizing lipid metabolism.

**Figure 3:**
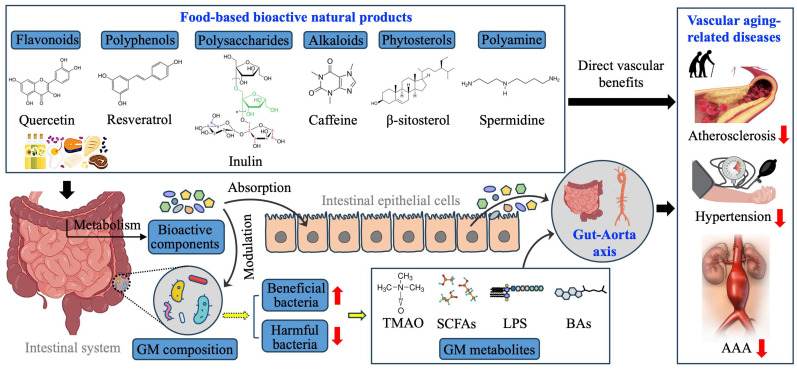
This model predicts how natural products regulate vascular aging-related diseases. Some food-based bioactive natural products could achieve direct vascular benefits in their prototype forms, whereas some are metabolized to active substances, which are then absorbed by intestinal epithelial cells and transported into circulating. The bioactive components cause GM alterations in either bacteria composition or metabolites or both, which contribute to gut-aorta axis.

Recent research underscores the critical interplay between GM and these natural products. After ingestion, compounds like dietary fiber, flavonoids, polyphenols, and alkaloids undergo metabolic transformations by GM, including fermentation, deglycosylation, oxidation, and conjugation. These processes yield secondary metabolites with modified bioactivity, which can influence their therapeutic efficacy. Furthermore, natural products contribute to maintaining microbial diversity, restoring GM balance, and enhancing nutrient absorption, indirectly promoting vascular health (Figure 3).

Given the emerging role of GM in vascular aging, ongoing studies aim to identify natural products that can modulate GM to delay age-related vascular changes and elucidate their underlying mechanisms. In the following, this review explores the potential of food-derived phytochemicals (Table 2: Chemical structure and food source of these natural products) to simultaneously impact GM and vascular health, emphasizing GM-targeted natural compounds as promising anti-vascular aging agents.

### 5.1. Flavonoids

Flavonoids, naturally occurring phenolic compounds found in fruits, vegetables, and plant-based foods, have been shown to offer numerous health benefits, particularly in cardiovascular health [154]. These compounds, present in foods like berries, citrus fruits, apples, onions, broccoli, green tea, cocoa, and red wine, include well-known flavonoids such as quercetin, epigallocatechin-3-gallate (EGCG), and kaempferol. Increasing evidence suggests that flavonoids can modulate GM, highlighting their potential as natural interventions against vascular aging through the GM. By targeting pathways such as SCFAs production, oxidative stress, and inflammation, flavonoids bridge the gut–vascular axis.

Quercetin, a well-studied flavanol, quercetin, is abundant in apples, berries, onions, citrus fruits, and leafy greens. It has prebiotic-like effects, promoting beneficial gut bacteria such as *Bifidobacterium* and *Lactobacillus* while inhibiting pathogens like *Escherichia coli* [155,156]. Quercetin enhances the production of SCFAs, reduces TMAO levels, and regulates BA metabolism [157,158,159]. These actions mitigate inflammation and oxidative stress, factors integral to vascular aging. Quercetin’s vascular benefits include alleviating endothelial dysfunction, reducing VSMCs senescence, and combating atherosclerosis by modulating microbial populations, such as increasing *Firmicutes* and decreasing *Verrucomicrobia* [160,161,162].

Epigallocatechin-3-Gallate (EGCG), predominantly found in green tea, cocoa, and grapes, alters GM associated with obesity and diabetes. It increases the *Firmicutes*-to-*Bacteroidetes* ratio, promotes beneficial bacteria like *Lactobacillus* and *Akkermansia*, and reduces harmful *Desulfovibrionaceae* [163,164]. By addressing chronic inflammation, oxidative stress, and endothelial dysfunction, common pathways in diabetes and vascular aging, EGCG aids vascular health. It enhances SCFA levels, inhibits vascular permeability via matrix metallopeptidase 9 (MMP-9) suppression, and improves endothelial function through the nuclear factor erythroid 2-related factor 2 (Nrf2)/cysteine-aspartic acid protease 3 (Caspase-3) pathway [165,166,167].

Kaempferol, present in apple skins, kale, spinach, celery, and broccoli, kaempferol restores gut flora balance in conditions like obesity, diabetes, and colitis. It reverses high-fat diet-induced changes in GM, such as increasing *Bacteroidetes* and reducing *Firmicutes*, and modulates metabolites linked to energy production and bile acid metabolism [168,169]. In vascular systems, kaempferol protects against oxidative and inflammatory damage via the Nrf2/heme oxygenase 1 (HO-1) pathway and prevents endothelial apoptosis through autophagy regulation via the phosphoinositide 3-kinase (PI3K)/protein kinase B (Akt)/mammalian target of rapamycin (mTOR) pathway [170,171].

### 5.2. Polyphenols

Polyphenols, including resveratrol, chlorogenic acid, ellagic acid (EA), ferulic acid, and secoisolariciresinol diglucoside (SDG), have garnered increasing attention for their potential role in managing vascular aging and CVDs, particularly through modulation of GM [172]. As vascular aging is characterized by increased arterial stiffness, which impairs the ability of arteries to expand and contract with pressure changes, polyphenols present a promising strategy to target GM-related metabolism and mitigate these age-related changes.

Resveratrol, a stilbene found in foods such as peanuts, grapes, and berries, has been shown to interact with GM to protect against aging and age-related CVDs [173,174,175]. Resveratrol supplementation increases populations of beneficial bacteria like *Akkermansia*, *Lactobacillus*, and *Bifidobacterium*, while reducing the *Firmicutes*-to-*Bacteroidetes* ratio and inhibiting the growth of *Enterococcus faecalis* [173,174,175,176,177,178]. Resveratrol also enhances SCFAs production, modulates BA metabolism, and reduces TMAO levels [179], all of which helps to reduce vascular inflammation and oxidative stress, crucial factors in vascular aging. Additionally, resveratrol directly benefits vascular health by enhancing NO production, upregulating endothelial nitric oxide synthase (eNOS), reducing endothelin-1 synthesis, inhibiting VSMCs proliferation, and protecting against arterial stiffness and vascular remodeling [180,181].

Chlorogenic acid, a phenolic acid found in coffee, apples, and blueberries, selectively modulates GM to improve metabolic functions [182,183,184,185]. In animal models, chlorogenic acid increases beneficial bacteria such as *Akkermansia* and enhances SCFA production [184,185]. Chlorogenic acid also activates the Nrf2 pathway in endothelial cells, improving endothelial function and reducing oxidative damage from oxLDL [186,187]. Furthermore, it exhibits antihypertensive effects and delays vascular senescence through the Nrf2/HO-1 pathway [188,189].

Ellagic acid (EA), found in berries, pomegranates, and nuts, influences GM composition by increasing *Lactobacillus* and reducing *Escherichia coli* populations [190]. EA is converted into bioavailable urolithins by GM [191], and these derivatives have been studied for their anti-aging and anti-CVD potential. EA and urolithins regulate VSMCs proliferation, endothelial cell dysfunction, lipid metabolism, and calcium handling, providing a multifaceted approach to preventing CVDs [192,193,194].

Ferulic acid, present in whole grains, fruits, vegetables, and coffee, acts as a prebiotic, promoting the growth of beneficial gut bacteria like *Olsenella* and *Faecalibaculum* while producing SCFAs [195]. Ferulic acid is also a potent antioxidant and anti-inflammatory agent that protects against endothelial dysfunction and may help lower blood pressure [196]. By modulating GM and lipid metabolism through the AMP-activated protein kinase α (AMPKα)/sterol regulatory element binding protein 1 (SREBP1)/acetyl-CoA carboxylase 1 (ACC1) pathway, ferulic acid has been shown to improve atherosclerotic injury in animal models [197].

Secoisolariciresinol diglucoside (SDG), found in flaxseeds and whole grains, has significant anti-inflammatory effects, partly attributed to its modulation of GM [198]. SDG influences the diversity and composition of GM, promoting bacteria that produce SCFAs while decreasing inflammation-related bacteria. It has been shown to slow the progression of atherosclerosis and suppress endothelial inflammation through inhibition of the Akt/IκB/NF-κB pathway [199].

### 5.3. Dietary Fiber

Dietary fiber, which is resistant to digestion in the upper gastrointestinal tract and undergoes fermentation in the colon, where it supports the expansion of beneficial microbes. This fermentation process promotes the growth of beneficial bacteria, such as *Bifidobacterium* and *Lactobacillus* species, which are known to contribute to gut health by supporting intestinal integrity and reducing inflammation, preventing CVDs and mitigating the effects of vascular aging.

Inulin is a type of dietary fiber found in plants like chicory root, Jerusalem artichoke, garlic, onions, asparagus, and bananas. In the colon, inulin is fermented by beneficial bacteria, producing SCFAs like butyrate [200]. These SCFAs help nourish colon cells, enhance intestinal barrier integrity, and exert anti-inflammatory effects. Additionally, inulin can restore microbiota balance and reduce microbiota–mucosa separation under high-fat diet conditions [201,202]. Studies also suggest that inulin improves vascular health, with effects such as improved endothelial function and reduced atherosclerotic lesions in animal models [203,204]. Inulin supplementation reverses endothelial dysfunction in mesenteric and carotid arteries by activating the NOS pathway, influenced by GM composition and BA production [205].

Pectin, found in the cell walls of fruits such as citrus, apples, and pears, is a polysaccharide rich in galacturonic acid. Although indigestible by human enzymes, pectin can be fermented by gut bacteria to produce SCFAs. Pectin supplementation influences GM diversity, stimulating the growth of beneficial bacteria like *Bacteroides* and *Eubacterium* [206,207]. Although research on pectin’s vascular effects is limited, recent studies suggest that high-esterified pectin supplementation can improve blood pressure, reduce heart lipid content, and regulate cardiac gene expression in rat models [208].

β-Glucan, a soluble fiber found in mushrooms, yeast, oats, and barley, has various health benefits, including antioxidant effects, blood sugar regulation, cholesterol reduction, and immune support [209]. β-Glucan consumption has been linked to increased levels of beneficial bacteria, including *Akkermansia*, *Lactobacillus*, and *Lachnospiraceae* [209]. Research indicates that β-glucan positively modulates vascular function in both animal models and human studies, improving outcomes such as blood pressure and GM composition [210,211,212,213,214]. In atherosclerotic mice and hypercholesterolemic human subjects, β-glucan supplementation decreased body weight, blood pressure, and modified GM, promoting the production of anti-inflammatory metabolites.

### 5.4. Alkaloids

Alkaloids are a group of nitrogen-containing compounds found in plants, fungi, bacteria, and marine organisms, with caffeine and capsaicin being notable examples. Research has explored their pharmacological effects on CVDs and, more recently, the role of GM in mediating these effects.

Caffeine, a widely consumed substance in coffee, tea, chocolate, and soft drinks, caffeine’s impact on GM is still under study, but it has been shown to influence gut motility and acid production [215,216]. Some studies suggest that caffeine increases the abundance of beneficial bacteria, such as *Faecalibacterium* and *Roseburia*, while decreasing harmful bacteria like *Erysipelatoclostridium* [217]. In metabolic syndrome mouse models, caffeine improved the profile of SCFAs and BA metabolism [218,219]. Caffeine has well-established vascular benefits, including enhancing endothelial function through NO production, and may protect against cognitive decline in older women with vascular disordersts anti-inflammatory, antioxidant, and cardioprotective effect [220,221,222].

Capsaicin, primarily found in chili peppers, is a widely used spice known for its analgesic properties. Studies show that it can alter GM composition, increasing the abundance of bacteria such as *Faecalibacterium*, *Akkermansia*, and *Roseburi* [223]. In addition to modulating GM, capsaicin has been linked to vascular health, with potential benefits in preventing vascular aging in diabetes by reducing EC senescence [224]. Capsaicin also protects against arterial calcification and improves endothelial function [225,226], suggesting its role in cardiovascular protection and anti-aging effects.

### 5.5. Phytosterols

Phytosterols are plant-derived steroids with a similar structure to cholesterol, primarily found in vegetable oils, nuts, seeds, and whole grains. They play a vital role in maintaining the stability of cellular membranes. Consumption of phytosterols helps lower LDL cholesterol by competing with cholesterol for absorption in the digestive system, making them popular ingredients in functional foods like margarine, spreads, and yogurt. Studies suggest that GM may prefer phytosterols over cholesterol as a substrate [227], which could influence GM composition and contribute to cardiovascular health.

β-Sitosterol, the most abundant phytosterol, is found in plant oils, nuts, seeds, and grains. It has been shown to support cardiovascular health by reducing atherosclerotic plaques in mice [228,229,230], improving gut health by increasing beneficial bacteria like *Bifidobacterium* and *Lactobacillus*, and decreasing harmful bacteria such as *Desulfovibrionaceae* [231,232]. β-Sitosterol also has direct vascular effects, inhibiting vascular smooth muscle cell proliferation through the AMPK and mTOR pathways [233].

Stigmasterol, found in legumes, nuts, whole grains, and vegetables, has emerged for its hepatoprotective effects, particularly by remodeling GM [234,235,236]. It alters GM diversity, increasing beneficial bacteria and improving the balance of regulatory T cells to CD8^+^ T cells [235]. Stigmasterol also improves liver function by modulating bile acid profiles and reversing gut dysbiosis induced by a high-fat diet [236]. Vascular protection from stigmasterol includes inhibiting the proliferation of smooth muscle cells and ECs through various signaling pathways, including the MAPK pathway [237].

Overall, the impact of food-based natural products on gut microbiota, their derived metabolites, and mechanisms associated with vascular aging is summarized in Table 3 [160,162,174,205,206,208,224,233,238,239,240,241,242,243,244,245,246,247,248]. Table 4 [177,197,208,214,249,250,251,252,253,254,255,256,257,258,259,260,261,262,263,264,265] provides an overview of their effects on cardiovascular diseases, including atherosclerosis, hypertension, and abdominal aortic aneurysm (AAA), through gut microbiota modulation. These findings highlight the promising potential of incorporating gut microbiota-targeted dietary strategies into therapeutic approaches for managing vascular aging and preventing cardiovascular diseases.

## 6. Conclusions and Perspectives

Vascular aging is a complex process shaped by inflammation, oxidative stress, mitochondrial dysfunction, and cellular senescence. Emerging research emphasizes the central role of GM in these processes, highlighting a dynamic gut–vascular axis that significantly influences cardiovascular health. Dysbiosis exacerbates vascular aging by inducing oxidative stress, inflammation, and endothelial dysfunction, mediated by metabolites such as TMAO, SCFAs, LPS, and BAs. These microbial factors accelerate arterial stiffening, atherosclerosis, and mitochondrial dysfunction, hallmark features of vascular aging. While substantial progress has been made, further studies are essential to deepen our understanding of the molecular pathways connecting GM to vascular aging. Recent findings underscore the need to explore additional mechanisms, including mitochondrial dysfunction, loss of proteostasis, cellular senescence, apoptosis, and necroptosis, as potential links. Furthermore, discovering novel microbiota-derived metabolites and their impacts on vascular health could open new therapeutic avenues to mitigate aging-related vascular decline.

Nutrient-derived natural products offer promising strategies for regulating GM and combating vascular aging, addressing the challenge of promoting cardiovascular health. Compounds such as flavonoids, polyphenols, dietary fibers, alkaloids, and phytosterols not only exert direct cardiovascular benefits through anti-inflammatory, antioxidant, and metabolic effects but also indirectly enhance vascular health by modulating GM composition and activity. The intricate interplay between these natural products and GM results in the production of bioactive metabolites, restoration of microbial diversity, and improved gut barrier function, thereby influencing vascular integrity and function. Future research should prioritize identifying novel GM-derived metabolites, modulated by nutrient-derived natural products, linked to vascular health and developing personalized interventions tailored to individual GM profiles, enabling precise dietary strategies to combat vascular aging effectively.

Despite the promising potential of GM-targeted interventions, several limitations and challenges must be addressed before these strategies can be effectively translated into clinical practice. One major hurdle is the significant interindividual variability in GM composition, which can influence the efficacy of probiotics, prebiotics, and nutrient-derived natural products. Factors such as genetics, age, diet, medication use, and disease status all contribute to differences in microbial communities, resulting in variable responses to the same therapeutic intervention [266]. For instance, a probiotic strain that promotes vascular benefits in one individual may be ineffective or even counterproductive in another due to differences in microbial ecology or metabolite profiles [267]. Additionally, the long-term effects and safety of chronic modulation of the microbiota remain unclear, particularly in elderly or immunocompromised populations. The complexity of host-microbiota interactions and the need for precise, personalized approaches underscore the importance of developing robust biomarkers and diagnostic tools to stratify patients and tailor interventions accordingly. Addressing these challenges will be critical for harnessing the full therapeutic potential of GM-based strategies in the prevention and treatment of vascular aging and related CVDs.

While this review provides a comprehensive synthesis of current evidence on the interplay between GM, vascular aging, and cardiovascular health, several limitations should be acknowledged. First, the scope of the literature reviewed is constrained by the availability of published studies, and certain aspects of gut–vascular interactions remain underexplored, particularly in human cohorts and aged animal models. Second, substantial heterogeneity exists in the methodologies used among the cited studies, including variations in experimental models, microbiota profiling techniques, and outcome measures, which may limit direct comparability and the ability to draw definitive conclusions. Third, potential publication bias, favoring studies with positive or significant results, may skew the overall understanding of the field. Additionally, variability in study populations, such as differences in age, sex, genetic background, dietary patterns, and environmental exposures, may influence the generalizability of findings. These factors should be considered when interpreting the current evidence and in designing future research to address these gaps.

## Figures and Tables

**Table 1:** Gut-origin microbes act as functional probiotics attenuating vascular aging-related diseases.

Category (Phylum)	Bacteria Strains	Athero-sclerosis	Hyper-tension	AAA	GM Metabolites	Study Model	Ref.
*Actinobacteria*	*Bif. bifidum* PRL 2010	•	°	°	°	Bacteria-fed ApoE^−/−^ mice	[98]
	*Bif. breve* Bb4	•	°	°	↓ TMAO	Choline chloride-fed C57BL/6J mice	[99]
	*Bif. breve* CECT7263	°	•	°	°	Spontaneously hypertensive rats	[100]
	*Bif. lactis* HN019	°	•	°	°	Hypertensive women	[101]
	*Bif. pseudocatenulatum* G4	•	°	°	↑ BAs	Cholesterol-enriched-diet-fed SD rats	[102]
	*Bif. longum* BB536	•	°	°	↑ BAs	Cholesterol-enriched-diet-fed SD rats	[102]
	*Bif. longum* CCFM 1077	•	°	°	↑ BAs	High-cholesterol-diet-fed SD rats	[103]
	*Bif. longum* BL1 and BL7	•	°	°	↓ TMAO	Choline chloride-fed C57BL/6J mice	[103]
*Firmicutes*	*Lb. plantarum* 299v	•	•	°	°	Humans with coronary artery disease	[104]
	*Lb. plantarum* ZDY04	•	°	°	↓ TMAO	1.3% choline-fed ApoE^−/−^ mice	[105]
	*Lb. plantarum* E680	•	°	°	°	High-fat emulsion-fed ICR mice	[106]
	*Lb. plantarum* ATCC 8014	•	°	°	°	Propylthiouracil & cholesterol-fed mice	[107]
	*Lb. rhamnosus* GG	•	°	°	°	HFD-fed ApoE^−/−^ mice	[108]
	*Lb. rhamnosus* GR-1	•	°	°	°	HFD-fed ApoE^−/−^ mice	[108]
	*Lb. fermentum* H9	•	°	°	°	HFD-fed SD rats	[109]
	*Lb. fermentum* CEC 5716	°	•	°	°	Spontaneously hypertensive rats	[110]
	*Lb. fermentum* CEC 5716	•	•	°	°	L-NAME-treated Wister rats	[111]
	*Lb. fermentum* NCIMB 5221	•	°	°	°	Zucker diabetic fatty rats	[112]
	*Lb. brevis* 119-2	•	°	°	°	HFD-fed SD rats	[113]
	*Lb. mucosae* DPC 6426	•	°	°	°	HFD-fed ApoE^−/−^ mice	[114]
	*Lb. acidophilus* ATCC 4356	•	°	°	↓ LPS	LPS-treated HUVECs	[115]
	*Lb. amylovorus* CP1563	•	•	°	°	HFD-fed C57BL/6J mice	[116]
	*Lb. casei* Shirota	•	•	°	°	HFD-fed ApoE^−/−^ mice	[117]
	*Lb. paracasei* NTU 101	•	•	°	°	Hyperlipidemic hamsters	[118]
	*Lb. johnsonii* La1	°	•	°	°	Bacteria-fed Wistar rats	[119]
	*Lb. jensenii* ATCC 25258	°	•	°	°	Spontaneously hypertensive rats	[120]
	*Lb. helveticus*	•	•	°	°	Hypertensive subjects	[121]
	*Lb. reuteri* CCFM8631	•	°	°	°	Paigen atherogenic diet-fed mice	[122]
	*Lb. reuteri* ADR-3	°	•	°	°	High-fructose-fed rats	[123]
	*E. aerogenes* ZDY01	•	°	°	↓ TMAO	1.3% choline-fed BALB/c mice	[124]
	*E. faecium* M-74	•	°	°	°	Healthy human subjects	[125]
	*E. faecalis* ATCC 19433	•	°	°	°	HFD-fed C57BL/6J mice	[126]
	*P. acidilactici* RO37	•	°	°	°	Female ApoE^−/−^ mice	[127]
	*F. prausnitzii*	•	°	°	↓ LPS	HFD-fed ApoE^−/−^ mice	[128]
*Bacteroidetes*	*B. vulgatus* and *B. dorei*	•	°	°	↓ LPS	Human patients and ApoE^−/−^ mice	[129]
*Verrucomicrobia*	*A. muciniphila*	•	°	°	↓ LPS	HFD-fed ApoE^−/−^ mice	[130]

Notes: • indicates Observed Effect; ° indicates Not Applicable; ↑ indicates Increase/Promote; ↓ indicates Decrease/Inhibit. *Bif.* indicates *Bifidobacterium*; *Lb*. indicates *Lactobacillu*; *E.* indicates *Enterobacter*; *P.* indicates *Pediococcus*; *F.* indicates *Faecalibacterium*; *B.* indicates *Bacteroides*; *A.* indicates *Akkermansia*; *St.* indicates *Streptococcus*. AAA indicates Abdominal Aortic Aneurysm; GM indicates Gut Microbiota; TMAO indicates Trimethylamine-N-oxide; LPS indicates Lipopolysaccharide; BAs indicates bile acids; SD indicates Sprague Dawley; HFD indicates High Fat Diet; Ref. indicates References.

**Table 2:** Chemical structure and food source of the mentioned natural products.

Category	Compounds	Chemical Structure	Food Source
Flavonoids	Quercetin	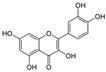	Apples, berries, onions, capers, citrus fruits, and leafy greens
	EGCG	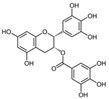	Grapes, wine, cocoa, apricots, beans, and green tea
	Kaempferol	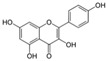	Apple skins, kale, spinach, celery, lettuce, and broccoli
Polyphenols	Resveratrol	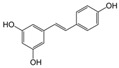	Peanuts, grapes, blueberries, raspberries, and mulberries
	Chlorogenic acid	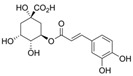	Coffee beans, apples, pears, blueberries, artichokes, tomatoes, and potatoes
	Ellagic acid	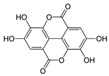	Berries, grapes, pomegranate, spinach, lettuce, walnuts, and pecans
	SDG	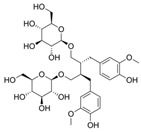	Flaxseeds, wheat bran, oats, barley, rye, and legumes
	Ferulic Acid	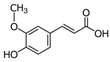	Wheat, oats, rice, corn, oranges, apple, cherries, tomato, onion, almonds, peanuts, flaxseeds, and coffee beans
Polysaccharides	Inulin	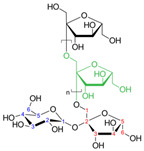	Chicory root, Jerusalem artichoke, garlic, onions, asparagus, and bananas
	Pectin (Galacturonic acid)	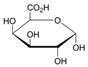	Citrus fruits, apples, berries, guava, quince, and pears
	β-glucan (Cellulose)	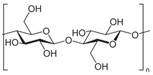	Oats, barley, whole grains, mushrooms, and yeast
Alkaloids	Caffeine	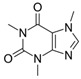	Coffee, tea, chocolate, and soft drinks
	Capsaicin	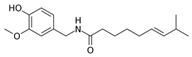	Chili peppers
Phytosterols	β-sitosterol	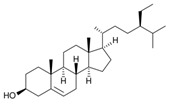	Plant-based oils, nuts, seeds, and whole grains
	Stigmasterol	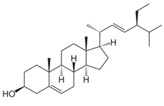	Soybeans, pistachios, oats, avocados, spinach
Polyamine	Spermidine	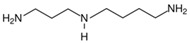	Wheat germ, soybeans, mushroom, corn, peas, and cheese

Notes: EGCG indicates Epigallocatechin-3-gallate; SDG indicates Secoisolariciresinol diglucoside.

**Table 3:** The impact of food-based natural products on gut microbiota modulation and vascular aging-associated factors.

Category	Compounds	GM Composition (↑↓)	GM Metabolites (↑↓)	I/O	VF	A/S	Mechanism of Actions	Refs.
Flavonoids	Quercetin	↑ *Bifidobacterium* ↑ *Lactobacillus* ↓ *Escherichia coli*	↑ SCFAs ↑ BAs ↓ TMAO	•	•	•	√ Induce apoptosis in VSMCs. √ Regulate TRAF6-MAPK pathway in EC.	[160,162]
	EGCG	↑ *Firmicutes* ↑ *Lactobacillus* ↓ *Desulfovibrionaceae*	↑ SCFAs	•	•	•	√ Suppress MMP-9 and VEGF activation. √ Activate Nrf2/Caspase-3 signaling.	[238]
	Kaempferol	↑ *Firmicutes* ↓ *Bacteroidetes*	↓ BAs	•	•	°	√ Regulate NF-κB signaling pathway. √ Inhibit PI3K/Akt/mTOR pathway.	[239]
Polyphenols	Resveratrol	↑ *Akkermansia* ↑ *Lactobacillus* ↑ *Bifidobacterium* ↓ *Enterococcus faecalis*	↑ SCFAs ↓ TMAO	•	•	•	√ Upregulate eNOS activity. √ Reduce endothelin-1 synthesis. √ Inhibit VSMC proliferation.	[174,240]
	Chlorogenic acid	↓ *Blautia* ↓ *Sutterella* ↑ *Dubosiella* ↑ *Romboutsia*	↑ SCFAs ↑ BAs	•	•	•	√ Improve Nrf2 activation. √ Regulate Rap1 and PI3K/AKT pathways. √ Regulate Nrf2/HO-1 pathway.	[241]
	Ellagic acid	↑ *Lactobacillus* ↓ *Escherichia coli* ↓ *Bacteroidetes* ↑ *Akkermansia*	°	•	•	•	√ Inhibit VSMC proliferation. √ Improve endothelial function. √ Modulate Ca^2+^ intake and release.	[242,243]
	SDG	↑ *Proteobacteria* ↑ *Roseburia* ↑ *Blautia*	↑ SCFAs	•	•	•	√ Inhibit the Akt/IκB/NF-κB pathway.	[244]
Polysaccharides	Inulin	↑ *Bifidobacterium* ↑ *Lactobacillus* ↓ *Firmicutes*	↑ SCFAs ↓ BAs	•	•	•	√ Activate NO synthase/NO pathway.	[241]
	Pectin	↑ *Firmicutes* ↑ *Eubacterium eligens*	↑ SCFAs	°	•	°	√ Reduce blood pressure.	[242,244]
Alkaloids	Caffeine	↑ *Faecalibacterium* ↑ *Roseburia* ↓ *Erysipelatoclostridium*	↑ SCFAs ↓ BAs	•	•	°	√ Increase NO production.	[245]
	Capsaicin	↑ *Faecalibacterium* ↑ *Akkermansia*	°	•	•	•	√ Regulate TRPV1/SIRT1 pathway. √ Upregulate SIRT6 pathway.	[224,246]
Phytosterols	β-sitosterol	↑ *Bifidobacterium* ↓ *Desulfovibrionaceae*	↓ TMAO	•	•	°	√ Activate AMPK pathway. √ Suppress mTOR pathway.	[233,247]
	Stigmasterol	↑ *Lactobacillus* ↓ *Erysipelatoclostridium*	°	•	•	°	√ Inactivate MAPK signaling. √ Induce arrest of cell cycle.	[248]

Notes: • indicates Observed Effect; ° indicates Not applicable; ↑ indicates Increase/Promote; ↓ indicates Decrease/Inhibit. GM indicates Gut Microbiota; I/O indicates Inflammation and Oxidative Stress; VF indicates Vascular Function; A/S indicates Aging/Senescence; TMAO indicates Trimethylamine-N-oxide; LPS indicates Lipopolysaccharide; BAs indicates bile acids; SCFAs indicates short chain fatty acids. Ref. indicates References.

**Table 4:** The impact of food-based natural products or extracts on cardiovascular disease by modulating gut microbiota.

Category	Compounds	Athero-sclerosis	Hyper-tension	AAA	GM Modulation (↑↓)	Study Model; Dosage	Ref.
Flavonoids	Quercetin	•	°	°	↓ *Verrocomicrobia* ↑ *Actinobacteria*, *Cyanobacteria and Firmicutes*	HFD-fed LDLR^−/−^ mice; 10 µg/day Quercetin	[249]
	EGCG	•	°	°	↑ *Roseburia*, *Rothia*, *Parabacteroides* and *Akkermansia* ↓ *Bilophila* and *Alistipes*	HFD-fed ApoE^−/−^ mice; 150 mg/kg/day Extract of Peanut Skin containing EGCG	[250]
	EGCG	°	•	°	↑ Paraprevotella and Bacteroides ↑ Allobaculum and Bifidobacterium	High salt diet-fed Wistar rats; 500 mg/kg/day Extract of Selenium-enriched and Ordinary Green Tea containing EGCG	[251]
	Kaempferol	•	°	°	↑ *Bacteroidetes*, *Verrucomicrobiota*, and *Akkermansiaceae*	HFD-fed ApoE^−/−^ mice; 6.2 mL/kg Dry Red Wine containing Kaempferol	[252]
	Kaempferol	°	•	°	↑ *Lactobacillus* and SCFAs ↓ Ratio of *Firmicutes*/*Bacteroidetes* ↓ *Clostridiaceae*	Spontaneously hypertensive rats; 0.9 g/kg Extracts of *Scutellaria baicalensis Georgi* and *Sophora japonica* L. containing Kaempferol	[253]
Polyphenols	Resveratrol	•	°	°	↑ *Lactobacillus* and *Bifidobacterium* ↓ TMAO and ileal BA	TMAO-induced atherosclerosis in ApoE^−/−^ mice; 0.4% Resveratrol	[177]
	Resveratrol	°	•	°	↓ *Bacteroidetes* ↑ *Verrucomicrobia* ↑ *Firmicutes to Proteobacteria ratio*	High-fructose diet-fed SD rats; 50 mg/L Resveratrol	[254]
	Resveratrol	°	•	°	↓ *Butyricicoccus* and Acetic acid ↑ *Actinobacteria*	0.5% adenine diet-fed SD rats; 10 mg/kg/day Resveratrol	[255]
	Chlorogenic acid	•	°	°	↑ *Mogibacteriaceae*, *Coprococcus*, *Dorea*, *Ruminococcus*, *Firmicutes*, and *Desulfovibrio*	HFD-fed ApoE^−/−^ mice; Extract of Green Coffee Bean, equivalent to 220 mg/kg of Chlorogenic acid	[256]
	Chlorogenic acid	°	•	°	↓ *Lachnospiraceae*, and *Oscillospira*	HFD-fed Wistar rats; 2 g Chlorogenic acid per kg of food	[257]
	Ellagic acid	°	•	°	↑ *Lactococcus* ↓ *Bifidobacterium*	Adult metabolic syndrome patients under secondary pharmacological prevention and without previous CVD events; 900 mg/day Pomegranate Extract containing Ellagic acid	[258]
	Ferulic acid	•	°	°	↓ *Fimicutes*, *Erysipelotrichaceae*, and *Ileibacterium*	HFD-fed ApoE^−/−^ mice; 40 mg/kg/day Ferulic Acid	[197]
Polysaccharides	Inulin	•	°	°	↑ Ratio of propionate to acetate	High cholesterol diet-fed ApoE^−/−^ mice; 10% inulin of diet weight	[259]
	Pectin	•	°	°	↑ SCFAs	High-fat/cholesterol diet-fed ApoE^−/−^ mice; 20% Pectin of diet weight	[260]
	Pectin	°	•	°	↑ *Bacteroides/Prevotella* ↑ *Lactobacillus*, and *Bifidobacterium* ↓ *Clostridium coccoides*	High sucrose diet-fed Wistar rats; 10% High-Esterified Pectin supplementation in diet	[208]
	β-glucan	•	°	°	↑ *Eisenbergiella* and *Romboutsia* ↑ SCFAs ↓ LPS	High-fat/high-cholesterol diet-fed LDLR^−/−^ mice; 0.8% Oat fiber containing 22% β-glucan	[261]
	β-glucan	°	•	°	↑ *Bacteroides* ↓ *Firmicutes*	Mildly hypercholesterolemic human subjects; 3 g/day High Molecular Weight Barley β-glucan	[214]
Alkaloids	Caffeine	°	•	°	↓ Ratio of *Firmicutes* to *Bacteroidetes*	High-carbohydrate/high-fat diet-fed Wistar rats; 5% Spent Coffee Grounds containing Caffeine	[262]
	Capsaicin	•	°	°	↑ *Turicibacter*, *Odoribacter*, and *Ileibacterium* ↓ Deoxycholic acid, cholic acid, hypoxanthine, and stercobilin	HFD-fed ApoE^−/−^ mice; 0.01% Capsaicin of diet weight	[263]
Phytosterols	β-sitosterol	•	°	°	↓ TMAO ↑ *Actinobacteriota*, *Bacteroidota*, *Desulfobacterota*, and *Firmicute* ↓ *Proteobacteria*, *Verrucomicrobiota*	High-choline diet-fed ApoE^−/−^ mice; 400 mg/kg/d β-sitosterol	[264]
Polyamine	Spermidine	°	°	•	↓ *Parabacteroides* ↑ *Prevotella*, *Desulfovibrionaceae*, *Campylobacterales*, and *Helicobacter*	Porcine Pancreatic Elastase-induced AAA model in C57BL/6 mice; 3 mM Spermidine via drinking water	[265]

Notes: • indicates Observed Effect; ° indicates Not applicable; ↑ indicates Increase/Promote; ↓ indicates Decrease/Inhibit. EGCG indicates Epigallocatechin-3-gallate; AAA indicates Abdominal Aortic Aneurysm; GM indicates Gut Microbiota; TMAO indicates Trimethylamine-N-oxide; LPS indicates Lipopolysaccharide; BAs indicates bile acids; SD indicates Sprague Dawley; HFD indicates High Fat Diet; Ref. indicates References.

## Abbreviations

BAs, bile acids; CMA, chaperone-mediated autophagy; CVDs, cardiovascular diseases; EA, ellagic acid; ECs, endothelial cells; EGCG, epigallocatechin-3-gallate; FMO, flavin mono-oxygenase; GM, gut microbiota; HFD, high fat diet; HSP, heat shock protein; IL, interleukin; LPS, lipopolysaccharides; MMP, matrix metalloproteinase; NO, nitric oxide; NOS, nitric oxide synthase; ROS, reactive oxygen species; SCFAs, short-chain fatty acids; SDG, secoisolariciresinol diglucoside; TMAO, trimethylamine-N-oxide; UPS, ubiquitin-proteasome system; VSMC, vascular smooth muscle cells.
